# Prognostic Value of Different Biological Age Estimation Methods in Breast Cancer Patients: A Population‐Based Cohort Study Using NHANES 1999–2020

**DOI:** 10.1002/hsr2.73238

**Published:** 2026-09-13

**Authors:** Guodong Chen, Wei Lin, Xueliang Cheng, Xin Chang

**Affiliations:** ^1^ Department of Breast Surgery The Second Hospital of Jilin University Changchun China; ^2^ Department of Spinal Surgery The Second Hospital of Jilin University Changchun China

**Keywords:** all‐cause mortality, biological age, breast cancer, national health and nutrition examination survey (NHANCE)

## Abstract

**Background and Aims:**

Breast cancer is the most common malignancy in women, and advanced biological ageing correlates with increased incidence. However, the prognostic value of comprehensive biological age biomarkers remains unclear. This study aimed to investigate the association between composite biological age indices and mortality in breast cancer patients.

**Methods:**

We analyzed data from 399 breast cancer participants in the National Health and Nutrition Examination Survey (1999–2020). Three biological age indicators were calculated using 12 blood‐based biomarkers: Phenotypic Age (PA), Homeostatic Dysregulation (HD), and the Klemera‐Doubal Method (KDM). Survey‐weighted Cox models estimated hazard ratios (HR) for all‐cause mortality. Restricted cubic splines assessed linearity, and time‐dependent receiver operating characteristic curves evaluated predictive performance.

**Results:**

During a median follow‐up of 6.67 years, 144 deaths occurred. In fully adjusted models, higher indices were associated with increased mortality risk: HD (HR = 1.465, 95% CI: 1.169–1.836), KDM (HR = 1.017, 95% CI: 1.004–1.030), and PA (HR = 1.080, 95% CI: 1.023–1.140). Spline analyses confirmed linear associations. ROC analyses revealed a time‐stratified predictive capacity, finding that HD performed best for short‐term prediction, whereas PA showed superior efficacy for mid‐ to long‐term prognosis.

**Conclusion:**

Accelerated biological ageing is an independent predictor of mortality in breast cancer patients. The distinct time‐specific utilities of HD and PA suggest that biological age indicators may guide personalized risk stratification and clinical management.

## 1 Introduction

Breast cancer is the most common malignant tumor in women worldwide, with a mortality rate of approximately 15% of all diagnosed cases [1, 2]. Although the survival rate of patients with early‐stage breast cancer has been significantly improved with advances in diagnostic and treatment technologies, the prognosis for patients with advanced breast cancer remains poor, the 5‐year survival rate is still less than 30% [3, 4, 5]. Therefore, it is crucial to identify novel indicators that can predict prognosis and guide intervention.

Aging is one of the most important risk factors for cancer, as the incidence of cancer is closely linked to increasing age, and breast cancer is no exception [6]. According to the latest American Cancer Society report, in the United States approximately 99% of breast cancer related deaths occur in women aged 40 years and older, and approximately 71% occur in women aged 60 years and older [7]. Moreover, in the context of the accelerating global ageing of population, exploring the role of ageing in cancer is of greater importance and urgency than ever before.

Notably, age does not accurately reflect the functional state of the human body or organs, because despite individuals having the same chronological age, their rates of ageing may differ [8]. Therefore, chronological age is not a perfect indicator of the ageing process. Recently, biological age has attracted increasing attention because it could better reflect an individual's functional status by integrating biomarker information [9]. Telomeres are highly specialized structures covering the ends of linear chromosomes, composed of repetitive DNA sequences. Telomere length shortens with each cell division, thus representing the most traditional biological marker of biological age [10]. Some studies have investigated the relationship between telomere length and breast cancer, finding that telomere shortening is associated with an increased risk of breast cancer and poorer prognosis [11]. However, telomere length captures only a single dimension of cellular senescence and currently lacks clinical accessibility due to methodological standardization challenges. Recently, more comprehensive composite biomarkers of biological age have been developed and validated, including Homeostatic Dysregulation (HD), the Klemera‐Doubal Method (KDM), and PhenoAge (PA) method. These innovative measures integrate multiple clinical and biochemical parameters to provide a more holistic assessment of biological aging processes [12, 13, 14]. Emerging evidence demonstrates significant associations between these composite biomarkers and breast cancer risk, particularly for PA [12, 15]. Despite these advances in understanding these comprehensive biological age biomarkers and their relationship with breast cancer incidence, their potential role in predicting prognosis and survival outcomes remains unexplored.

Therefore, this study aims to utilize epidemiological data from National Health and Nutrition Examination Survey (NHANES) 1999–2020 to investigate the relationship between three comprehensive biological age biomarkers (HD, KDM, and PA) and the prognosis of breast cancer patients.

## 2 Methods

### 2.1 Study Population

**Figure 1:**
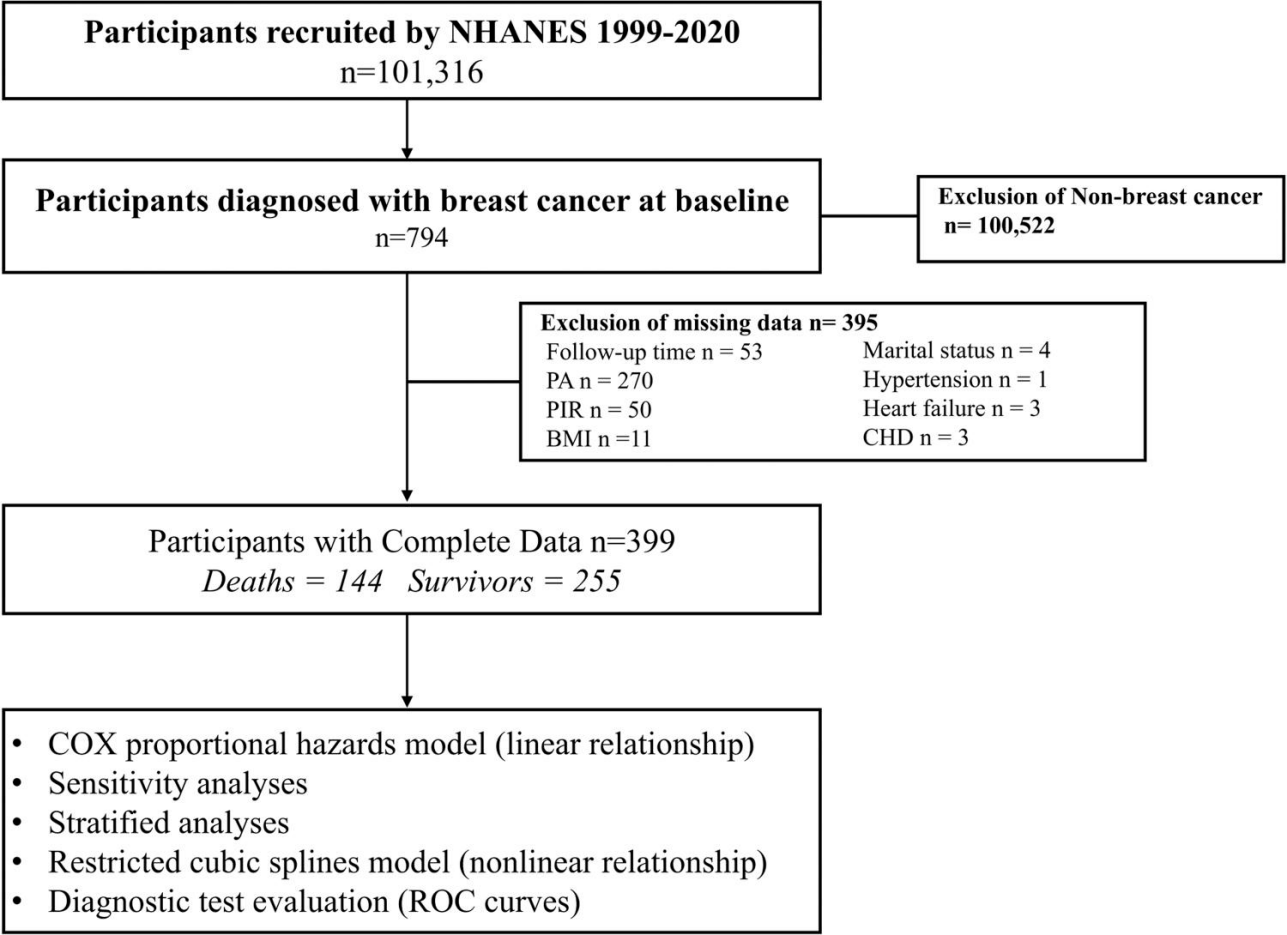
Study design diagram. From NHANES 1999–2020, 101,316 participants were screened. After excluding participants without breast cancer at baseline (*n* = 100,522), 794 participants remained. Additional exclusions for missing follow‐up time, PA data, PIR, BMI, marital status, hypertension, heart failure, or CHD yielded 399 complete‐case participants, including 144 deaths and 255 survivors. Analyses included Cox models, sensitivity/subgroup analyses, restricted cubic splines, and time‐dependent ROC analyses. BMI, body mass index; CHD, coronary heart disease; NHANES, National Health and Nutrition Examination Survey; PA, phenotypic age; PIR, poverty income ratio; ROC, receiver operating characteristic.

The NHANES is a long‐term, large‐scale program led by the National Center for Health Statistics (NCHS), a division of the U.S. Centers for Disease Control and Prevention (CDC). Its primary objective is to collect comprehensive information on the health, nutrition, and lifestyle of the general U.S. population. The NHANES protocol has received ethical approval from the NCHS Research Ethics Review Board, with ongoing annual reviews (Protocol #98–12, Protocol #2005–06, Continuation of Protocol #2005–06, Continuation of Protocol #2011–17, and Protocol #2018–01). Data collection methods include interviews, physical examinations, and laboratory tests, which are conducted either at mobile examination centers (MEC) or in participants' homes [16]. Detailed information on recruitment, study design, population characteristics, and data collection procedures is available from the Centers for Disease Control and Prevention (https://www.cdc.gov/nchs/nhanes/index.htm). For this population‐based cohort analysis, we used NHANES continuous cycles spanning 1999–2020 linked to mortality follow‐up data. A total of 399 participants were included based on the following criteria: a history of breast cancer, complete data for all components of the biological age prediction models, available mortality follow‐up, valid survey design variables, and complete covariate information (Figure 1). Breast cancer survivors were identified from the NHANES Medical Conditions Questionnaire as participants who self‐reported that they had ever been told by a doctor or other health professional that they had cancer and reported breast cancer as the cancer type. Eligible participants were required to have available mortality follow‐up, complete biomarker data for calculating HD, KDM, and PA, valid survey design variables, and complete covariate information; participants missing any of these data were excluded.

### 2.2 Exposure and Outcome Assessment

Three biological age indicators—HD, KDM, and PA—were calculated using a set of 12 selected blood chemistry parameters: albumin, alkaline phosphatase, C‐reactive protein, total cholesterol, creatinine, glycated hemoglobin, systolic blood pressure, blood urea nitrogen, uric acid, lymphocyte percentage, mean cell volume, and white blood cell count. These algorithms were developed and standardized using data from NHANES III (1988–1994), and have been described in detail by Nakazato et al. [17], Klemera et al. [18], and Levine et al. [16]. The corresponding R code is publicly available via the “BioAge” package at https://github.com/dayoonkwon/BioAge. HD, KDM, and PA were calculated using the BioAge R package according to the published algorithms for homeostatic dysregulation, the Klemera‐Doubal method, and Phenotypic Age, respectively [16, 18, 19]. All‐cause mortality was defined using NHANES‐linked National Death Index data; participants were followed from the NHANES examination date until death or censoring at the last available mortality follow‐up, and those without valid follow‐up time were excluded. Mortality follow‐up data were obtained from the National Death Index, provided by the U.S. CDC. All participants (except *n* = 15 with missing follow‐up time) had information on mortality status and person‐months of follow‐up. Mortality status and follow‐up time in person‐months were used to define all‐cause mortality and survival time. The median follow‐up time for the included population was 6.67 years, with an interquartile range of 7.42 years.

### 2.3 Covariate Assessment

Potential confounders considered in the analysis included age, race/ethnicity, body mass index (BMI, kg/m^2^), smoking status, educational attainment, marital status, poverty‐income ratio (PIR), and self‐reported chronic heart disease (CHD), hypertension, diabetes, and heart failure. Race/ethnicity was summarized in five categories for descriptive analyses and was modeled as non‐Hispanic White versus other race/ethnicity in Cox regression models because of limited subgroup sample sizes. Smoking status was defined as current cigarette smoking based on SMQ020 and SMQ040; hypertension, diabetes, heart failure, and CHD were defined by self‐reported physician diagnosis using BPQ020, DIQ010, MCQ160B, and MCQ160C, respectively. Race/ethnicity was derived from NHANES race/Hispanic‐origin recodes and was dichotomized as non‐Hispanic White versus other groups in Cox models to reduce sparse‐data instability, while the original five‐category variable was retained for descriptive analyses. BMI was calculated as weight in kilograms divided by height in meters squared.

### 2.4 Statistical Analysis

All statistical analyses were pre‐specified in accordance with the Strengthening the Reporting of Observational Studies in Epidemiology (STROBE) guidelines for cohort studies [20]; detailed reporting can be found in Supporting Information S1: Table S1. All primary analyses accounted for the complex multistage sampling design of NHANES using MEC examination weights, strata, and primary sampling units. For the combined 1999–March 2020 analysis, 4‐year MEC examination weights were used for the 1999–2002 cycles, 2‐year MEC examination weights were used for the 2003–2016 cycles, and the special 2017–March 2020 pre‐pandemic MEC weights were used for the final period; these weights were rescaled according to the duration represented by each period to construct 21.2‐year analytic weights [21]. At baseline, continuous variables were summarized as survey‐weighted means and standard deviations, and categorical variables were summarized as unweighted counts with survey‐weighted percentages. *p* values for baseline comparisons were calculated using survey design‐based tests, with Rao–Scott adjusted *χ* ^2^ tests used for categorical variables.

Survey‐weighted Cox proportional hazards models were used to estimate hazard ratios (HRs) and 95% confidence intervals (CIs) for the associations of HD, KDM, and PA with all‐cause mortality in breast cancer participants. Three progressively adjusted models were constructed: Model 1 adjusted for age, non‐Hispanic White race/ethnicity, and PIR; Model 2 additionally adjusted for BMI, educational attainment, marital status, and smoking status; and Model 3 further adjusted for hypertension, diabetes, heart failure, and CHD. Covariates were selected based on previous studies of mortality among breast cancer or cancer survivors and their clinical relevance, covering demographic factors, socioeconomic status, adiposity, smoking status, and major cardiometabolic comorbidities [22, 23, 24]. HD, KDM, and PA were entered into Cox models as unstandardized continuous variables; therefore, HRs represent the mortality risk associated with each one‐unit increase in the corresponding biological age indicator. The proportional hazards assumption was evaluated using Schoenfeld residuals in Cox models constructed with the same covariate structure as the main models.

Restricted cubic spline (RCS) analyses were conducted within survey‐weighted Cox models to assess potential nonlinear dose–response relationships. Three knots were placed at the 10th, 50th, and 90th percentiles of each biological age indicator, and the median value was used as the reference. *p* values for overall association and nonlinearity were calculated for each RCS model.

Exploratory time‐dependent receiver operating characteristic (ROC) analyses were performed to compare the discriminatory performance of PA, HD, and KDM across 1‐ to 10‐year prediction horizons. Because follow‐up time was recorded in months, yearly prediction horizons were converted to months for the time‐dependent ROC analyses. Time‐dependent AUCs were estimated using the Kaplan–Meier method.

Sensitivity analyses were conducted to assess the robustness of the main findings. These included unweighted Cox models, survey‐weighted Cox models after excluding biomarker‐specific outliers greater than 3 standard deviations, and survey‐weighted Cox models additionally adjusted for estrogen use. Exploratory categorical analyses compared participants in the highest tertile of each biological age indicator with those in the lower two tertiles combined. Three‐level tertile Cox models were not retained because the lowest tertile of PA contained no death events, resulting in unstable estimates.

All statistical analyses were performed using R software (version 4.2.3). The key R packages used in the main analyses included survey, survival, Hmisc, gtsummary, and survivalROC. All statistical tests were two‐sided, with *p* < 0.05 considered statistically significant.

## 3 Results

### 3.1 Baseline Characteristics of Participants

As shown in Table 1, a total of 399 breast cancer participants were included in the survey‐weighted analysis, including 255 survivors and 144 deceased participants. The survey‐weighted mean age was 64.7 years overall, 61.2 years among survivors, and 74.2 years among deceased participants (*p* < 0.001). Race/ethnicity did not differ significantly by mortality status (*p* = 0.54), and non‐Hispanic White participants accounted for most of the weighted study population. Compared with survivors, deceased participants had a lower PIR, lower BMI, lower educational attainment, and a higher proportion of being widowed, divorced, or separated. The prevalence of smoking, hypertension, diabetes, heart failure, and CHD was also higher among deceased participants. All three biological aging markers PA, HD, and KDM were significantly higher in deceased participants than in survivors (*p* < 0.001 for all).

**Table 1:** Baseline characteristics of included participants.

Characteristic	Overall *N* = 399	Survive *N* = 255	Death *N* = 144	*p* value
Age, years	64.706 (12.131)	61.182 (11.326)	74.246 (8.610)	*p* < 0.001
Race/ethnicity				*p* = 0.54
Mexican American	41 (2.7%)	33 (3.3%)	8 (1.3%)	
Other Hispanic	18 (1.4%)	16 (1.6%)	2 (0.9%)	
Non‐Hispanic White	264 (86.5%)	154 (86.3%)	110 (86.9%)	
Non‐Hispanic Black	53 (5.7%)	34 (5.2%)	19 (7.0%)	
Other race	23 (3.7%)	18 (3.7%)	5 (4.0%)	
PIR	3.314 (1.563)	3.643 (1.457)	2.422 (1.495)	*p* < 0.001
BMI, kg/m^2^	28.456 (6.696)	28.940 (6.547)	27.145 (6.942)	*p* = 0.007
Education level				*p* < 0.001
Less than 9th grade	34 (3.6%)	21 (2.3%)	13 (7.3%)	
9–11th grade	56 (10.2%)	24 (6.2%)	32 (21.1%)	
High school grad/GED or equivalent	90 (23.0%)	59 (22.7%)	31 (23.9%)	
Some college or AA degree	117 (27.4%)	78 (28.2%)	39 (25.4%)	
College graduate or above	102 (35.7%)	73 (40.6%)	29 (22.4%)	
Marital status				*p* = 0.002
Married or living with partner	204 (57.0%)	148 (63.1%)	56 (40.6%)	
Widowed, divorced or separated	177 (38.2%)	94 (31.9%)	83 (55.3%)	
Never married	18 (4.8%)	13 (5.0%)	5 (4.1%)	
Smoking	46 (9.9%)	23 (7.5%)	23 (16.6%)	*p* = 0.01
Hypertension	212 (44.5%)	118 (37.5%)	94 (63.4%)	*p* < 0.001
Diabetes	67 (13.0%)	32 (9.5%)	35 (22.4%)	*p* = 0.007
Heart failure	21 (3.8%)	7 (2.2%)	14 (7.9%)	*p* = 0.01
CHD	24 (5.9%)	4 (3.0%)	20 (13.8%)	*p* = 0.04
PA	61.044 (13.647)	56.744 (12.280)	72.683 (9.863)	*p* < 0.001
HD	2.516 (0.912)	2.292 (0.738)	3.121 (1.053)	*p* < 0.001
KDM	56.491 (16.360)	52.711 (13.954)	66.720 (17.989)	*p* < 0.001

*Note: p* values were determined based on variable type. Specifically, Student's *t*‐test was used for continuous variables, and Pearson's *χ* ^2^ test was used for categorical variables.

Abbreviations: BMI, body mass index; CHD, coronary heart disease; HD, homeostatic dysregulation; KDM, Klemera–Doubal method; PA, phenotypic age; PIR, poverty income ratio.

### 3.2 Associations Between Biological Aging Markers and Mortality

In survey‐weighted Cox proportional hazards models, HD, KDM, and PA were all associated with increased all‐cause mortality among breast cancer participants (Table 2). In the fully adjusted Model 3, each one‐unit increase in HD was associated with a higher risk of death (HR = 1.465, 95% CI: 1.169–1.836, *p* < 0.001). Similarly, each one‐unit increase in KDM was associated with increased mortality risk (HR = 1.017, 95% CI: 1.004–1.030, *p* = 0.009), and each one‐unit increase in PA was also associated with increased mortality risk (HR = 1.080, 95% CI: 1.023–1.140, *p* = 0.006).

**Table 2:** Cox regression analysis of the association between aging biomarkers and all‐cause mortality.

Biomarker	Model	*N*	Events	HR (95% CI)	*p* value
HD	Model 1	399	144	1.439 (1.122–1.846)	0.004
HD	Model 2	399	144	1.462 (1.161–1.841)	0.001
HD	Model 3	399	144	1.465 (1.169–1.836)	< 0.001
KDM	Model 1	399	144	1.015 (1.001–1.029)	0.03
KDM	Model 2	399	144	1.016 (1.003–1.030)	0.01
KDM	Model 3	399	144	1.017 (1.004–1.030)	0.009
PA	Model 1	399	144	1.086 (1.031–1.144)	0.002
PA	Model 2	399	144	1.077 (1.023–1.134)	0.005
PA	Model 3	399	144	1.080 (1.023–1.140)	0.006

*Note:* Model 1 adjusted for age, non‐Hispanic White race/ethnicity, and PIR; Model 2 additionally adjusted for BMI, educational attainment, marital status, and smoking status; Model 3 further adjusted for hypertension, diabetes, heart failure, and CHD. All models accounted for the complex sampling design of NHANES using MEC examination weights, strata, and primary sampling units.

### 3.3 Restricted Cubic Spline Analyses and Proportional Hazards Assumption

**Figure 2:**
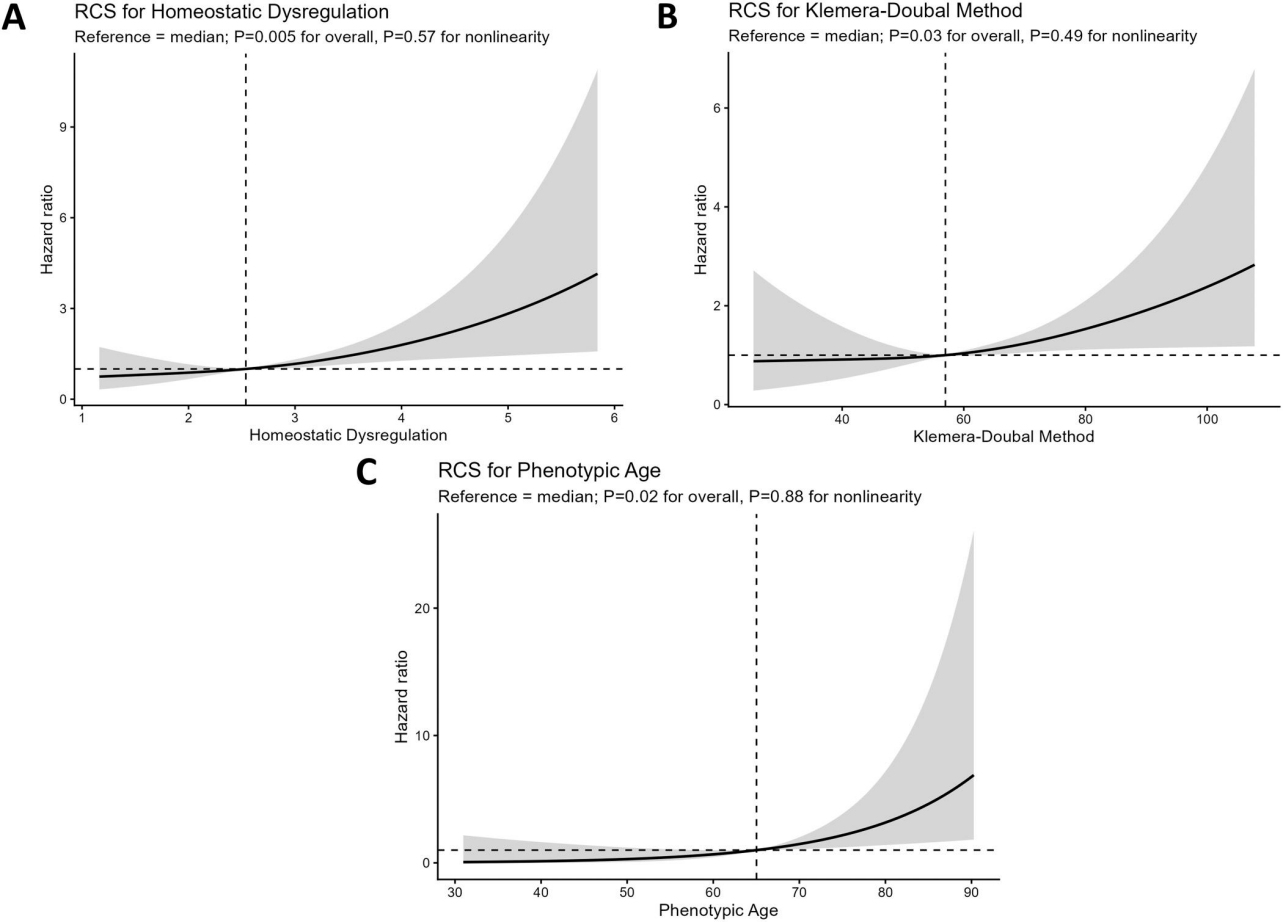
Survey‐weighted restricted cubic spline analyses of biological age indicators and all‐cause mortality. Restricted cubic spline plots for homeostatic dysregulation (A), Klemera–Doubal method biological age (B), and phenotypic age (C). Solid curves and shaded areas indicate hazard ratios and 95% confidence intervals. Vertical dashed lines mark median reference values; horizontal dashed lines mark HR = 1.0. Knots were at the 10th, 50th, and 90th percentiles. Models adjusted for age, race/ethnicity, poverty income ratio, BMI, education, marital status, smoking, hypertension, diabetes, heart failure, and coronary heart disease.

Survey‐weighted RCS analyses showed significant overall associations of HD, KDM, and PA with all‐cause mortality (Figure 2A–C). The overall association was statistically significant for HD (*p* = 0.005), KDM (*p* = 0.03), and PA (*p* = 0.02). No significant evidence of nonlinearity was observed for HD (*p* for nonlinearity = 0.57), KDM (*p* for nonlinearity = 0.49), or PA (*p* for nonlinearity = 0.88), suggesting generally monotonic associations across the observed biomarker distributions.

Schoenfeld residual tests showed no evidence of non‐proportionality for HD or KDM across adjusted models. As shown in Supporting Information S1: Table S2, for PA, the exposure‐specific tests suggested modest non‐proportionality in Models 2 and 3, although the global test was not significant in the fully adjusted model. Therefore, PA‐related Cox estimates were interpreted with caution.

### 3.4 Sensitivity Analyses

Sensitivity analyses showed generally consistent results (Supporting Information S1: Tables S3–S5). In unweighted Cox models, the associations of HD, KDM, and PA with all‐cause mortality remained directionally consistent with the survey‐weighted main analyses. After excluding biomarker‐specific outliers greater than 3 standard deviations, HD, KDM, and PA remained associated with all‐cause mortality in the fully adjusted survey‐weighted models. Additional adjustment for estrogen use yielded similar estimates, supporting the robustness of the main findings. In exploratory categorical analyses comparing the highest tertile with the combined lower two tertiles, high HD remained significantly associated with mortality, whereas the corresponding associations for KDM and PA were not statistically significant (Supporting Information S1: Table S6).

### 3.5 Predictive Performance of Aging Markers

**Figure 3:**
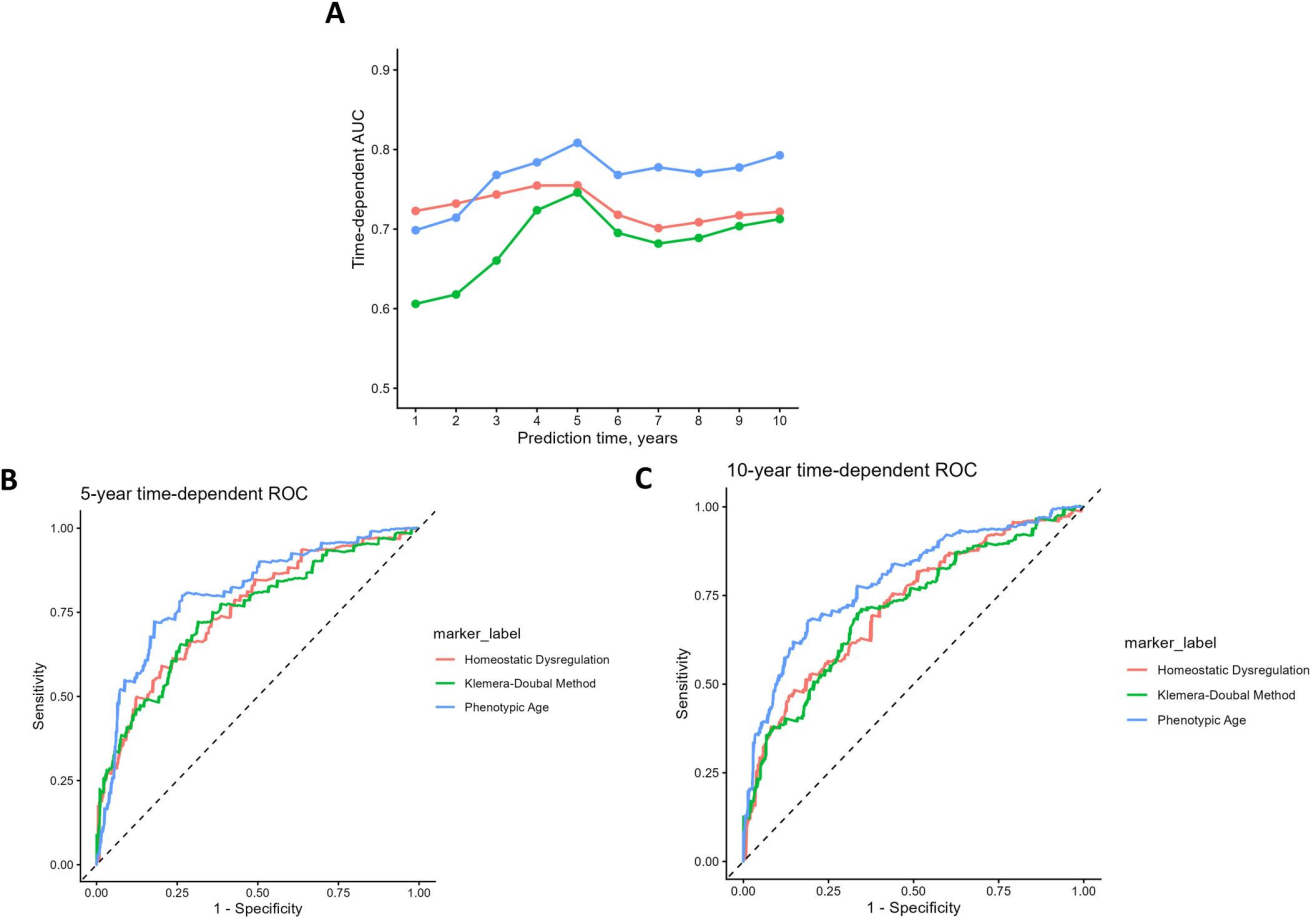
Exploratory time‐dependent ROC analyses of biological age indicators for all‐cause mortality. (A) AUC values for HD, KDM, and PA across 1–10 years of follow‐up. (B) ROC curves at the 5‐year time horizons. (C) ROC curves at the 10‐year time horizons. Red, green, and blue lines represent HD, KDM, and PA, respectively. The diagonal dashed line indicates no discrimination. AUCs were estimated using the Kaplan–Meier method. At 5 and 10 years, AUCs were 0.755/0.722 for HD, 0.746/0.713 for KDM, and 0.808/0.793 for PA. Abbreviations: AUC, area under the curve; HD, homeostatic dysregulation; KDM, Klemera–Doubal method; PA, phenotypic age.

Exploratory time‐dependent ROC analyses were performed to compare the discriminatory performance of HD, KDM, and PA for all‐cause mortality (Figure 3). HD showed slightly higher AUCs at the earliest prediction time points of 1 and 2 years. From 3 years onward, PA demonstrated the highest AUCs among the three biological age indicators across most prediction time points. At 5 years, the AUCs were 0.808 for PA, 0.755 for HD, and 0.746 for KDM. At 10 years, the corresponding AUCs were 0.793 for PA, 0.722 for HD, and 0.713 for KDM.

## 4 Discussion

There is a very close link between ageing and the development of cancers, particularly breast cancer [25, 26, 27]. Individuals of the same age may exhibit vastly different biological ageing states due to differences in genetic background, lifestyle, or environmental exposure [8]. In contrast, composite biomarkers of biological age, which integrate multiple clinical and biochemical parameters, can more accurately reflect an individual's actual degree of ageing [9]. In this study, we utilize large‐scale survey data from NHANES 1999–2020 to explore the relationship between comprehensive biological age biomarkers and breast cancer prognosis. The results showed that increases in the biological age indicators HD, KDM, and PA were all associated with an increase in all‐cause mortality in breast cancer patients, and after adjusting for factors such as chronological age, gender, and disease conditions, the results remained robust.

Biological age measurement does not have a gold standard. Different biological age indicators are weakly correlated with each other, so they can explain different dimensions of biological ageing [28, 29]. However, the combination of information from multiple clinical and biochemical parameters as indicators of biological ageing could better reflect a person's overall physiological state [12, 13]. To our knowledge, this is the first study to use comprehensive biological age biomarkers to predict the prognosis of breast cancer. We separately evaluated three comprehensive biological age biomarkers, including HD, KDM, and PA. These biomarkers are constructed based on different algorithms and theoretically differ in their assessment dimensions of biological ageing. However, the results consistently revealed that all three were positively correlated with a significantly increased risk of all‐cause mortality in breast cancer patients. Notably, stratified analyses revealed that, in contrast to HD and KDM, which exhibited significant associations with all‐cause mortality risk exclusively among non‐Hispanic white breast cancer patients, PA demonstrated robust and consistent associations with elevated all‐cause mortality risk across all racial subgroups, including non‐Hispanic whites, Hispanics, and other racial groups. This suggests that the multi‐system functional parameters integrated into the PA construct may capture a more fundamental biological ageing process, and therefore have general applicability across populations with different genetic backgrounds.

Through diagnostic test evaluation, our study found that these comprehensive biological age biomarkers showed significant time‐stratified differences in predicting all‐cause mortality from breast cancer. PA is more effective in predicting mid to long‐term prognosis, while HD is more effective in predicting short‐term prognosis. From the perspective of indicator composition, HD primarily includes immediate physiological function parameters that are routinely tested in clinical practice, such as systolic blood pressure, fasting blood glucose, creatinine, CRP, etc. [19]. These indicators reflect the immediate status of the human body's core systems, including circulation, metabolism, liver and kidney function, and immunity, at the time of assessment. Accordingly, elevated HD may more directly reflect the severity of short‐term physical impairment and therefore be more sensitive in predicting short‐term mortality events such as acute progression or treatment complications of breast cancer. On the other hand, PA integrates composite biological exposure factors related to long‐term cumulative ageing effects, such as chronological age, inflammatory factors, and metabolic markers [30]. So, it may explain why it is more effective in mid to long‐term prognosis assessment. In summary, our study findings suggest that in clinical practice, appropriate biological age biomarkers can be selected for testing at different follow‐up time points to optimize the comprehensive precision management of breast cancer patients. These exploratory findings suggest that the relative prognostic performance of different biological age indicators may vary across prediction horizons; however, their clinical utility for guiding follow‐up strategies requires further validation.

Our study successfully investigates the association between composite biological age biomarkers and breast cancer prognosis. Importantly, we reveal a time‐stratified predictive capacity. HD serves as a superior predictor for short term mortality risk, whereas PA exhibits greater efficacy for mid to long term prognosis assessment. This time‐stratified pattern suggests that biological age indicators may have potential value for risk stratification across different follow‐up periods, although their clinical utility for precision management requires further validation. Furthermore, PA appeared to show relatively consistent associations across racial and ethnic subgroups; however, these subgroup findings should be interpreted cautiously because of the limited subgroup sample sizes and the dichotomization of race/ethnicity in multivariable models. This provides a potentially universally applicable indicator for risk stratification in breast cancer survivors. Nevertheless, several limitations should be acknowledged. First, although NHANES provides nationally representative health examination data, it does not include detailed tumor‐specific information for breast cancer participants, such as TNM stage, histological grade, ER/PR/HER2 receptor status, Ki‐67 index, recurrence or metastasis status, or treatment regimens. Therefore, we were unable to evaluate whether biological age indicators provide incremental prognostic value beyond established tumor‐based prognostic systems. These metrics should not be interpreted as replacements for standard clinical prognostic factors, but rather as potential complementary indicators of host‐level physiological vulnerability that require validation in cohorts with detailed oncological data. Second, the predominantly non‐Hispanic White composition of the weighted study population may limit the generalizability of our findings to more diverse racial and ethnic groups. Third, NHANES participants were required to attend mobile examination centers, which may have excluded the frailest patients or those with advanced‐stage disease, potentially introducing healthy survivor bias. Excluding participants with missing data may have introduced selection bias and reduced sample representativeness. Fourth, biological age indicators were calculated using biomarkers measured at a single NHANES examination. Consequently, we could not assess longitudinal changes in biological aging during cancer treatment, after treatment, or during disease progression. Future studies with repeated biomarker measurements and detailed tumor and treatment information are needed to confirm these findings and clarify the clinical utility of biological age indicators in breast cancer prognosis.

## 5 Conclusions

This study demonstrates that accelerated biological ageing, as quantified by composite biomarkers (HD, KDM, and PA), is associated with an increased risk of all‐cause mortality in breast cancer patients, independent of chronological age, sex, and other common cancer risk factors. Importantly, our findings reveal a time‐stratified predictive capacity. HD serves as a superior predictor for short term mortality risk, whereas PA exhibits greater efficacy for mid to long term prognosis assessment. Furthermore, unlike HD and KDM, which were limited to non‐Hispanic White participants, PA maintained robust predictive value across diverse racial and ethnic subgroups. These findings suggest that composite biological age indicators may provide additional prognostic information in breast cancer survivors, but external validation in cohorts with detailed tumor and treatment data is needed before clinical application.

## Author Contributions

**Guodong Chen:** conceptualization, formal analysis, writing – original draft, methodology, software. **Wei Lin:** methodology, software, formal analysis, project administration, investigation. **Xueliang Cheng:** writing – review and editing. **Xin Chang:** writing – review and editing, data curation, supervision, validation.

## Funding

The authors have nothing to report.

## Ethics Statement

The CDC/NCHS Research Ethics Review Board granted approval for the detailed methods and protocols employed in the NHANES study. The data is publicly available, ethical approval was not required, and all data have been anonymized.

## Conflicts of Interest

The authors declare no conflicts of interest.

## Transparency Statement

The corresponding author (Xin Chang) affirms that this manuscript is an honest, accurate, and transparent account of the study being reported; that no important aspects of the study have been omitted; and that any discrepancies from the study as planned have been explained.

## Supporting information


Supporting File

## Data Availability

All data can be downloaded from the NHANES website (https://wwwn.cdc.gov/nchs/nhanes/Default.aspx).
