# Supplementary material for: Prognostic Value of Different Biological Age Estimation Methods in Breast Cancer Patients: A Population‐Based Cohort Study Using NHANES 1999–2020
Source: Health Sci Rep. 2026 Sep 13;9(9):e73238. doi: 10.1002/hsr2.73238 (PMC13572853; doi:10.1002/hsr2.73238)
Supplement: Supplementary file 1 [file HSR2-9-e73238-s001.docx]

**Supplementary Table 1. STROBE Checklist**

|  | **Item No** | **Recommendation** | **Section/ Exhibit** |  |  |
| --- | --- | --- | --- | --- | --- |
| **Title and abstract** | 1 | (*a*) Indicate the study’s design with a commonly used term in the title or the abstract | Title: Prognostic Value of Different Biological Age Estimation Methods in Breast Cancer Patients: A Population-Based Cohort Study Using NHANES 1999–2018 |  |  |
|  |  | (*b*) Provide in the abstract an informative and balanced summary of what was done and what was found | Abstract: Our findings indicate that accelerated biological ageing may be associated with an increased risk of all-cause mortality in breast cancer patients, suggesting that slowing biological ageing may improve prognosis in breast cancer patients, thereby contributing to the clinical management of breast cancer. |  |  |
| **Introduction** | | |  |  |  |
| Background/rationale | 2 | Explain the scientific background and rationale for the investigation being reported | Introduction: Moreover, in the context of the accelerating global ageing of population, exploring the role of ageing in cancer is of greater importance and urgency than ever before. Notably, age does not accurately reflect the functional state of the human body or organs, because despite individuals having the same chronological age, their rates of ageing may differ…… |  |  |
| Objectives | 3 | State specific objectives, including any prespecified hypotheses | This study aimed to compare the prognostic value of HD, KDM, and PA for all-cause mortality among breast cancer participants in NHANES 1999–2018. |  |  |
| **Methods** | | |  |  |  |
| Study design | 4 | Present key elements of study design early in the paper | Fig1 |  |  |
| Setting | 5 | Describe the setting, locations, and relevant dates, including periods of recruitment, exposure, follow-up, and data collection | Methods: Study Population |  |  |
| Participants | 6 | (*a*) Give the eligibility criteria, and the sources and methods of selection of participants. Describe methods of follow-up | Methods: Study Population, Figure 1 |  |  |
|  |  | (*b*) For matched studies, give matching criteria and number of exposed and unexposed | Not applicable. |  |  |
| Variables | 7 | Clearly define all outcomes, exposures, predictors, potential confounders, and effect modifiers. Give diagnostic criteria, if applicable | Methods: Exposure and Outcome Assessment, Covariate Assessment |  |  |
| Data sources/ measurement | 8* | For each variable of interest, give sources of data and details of methods of assessment (measurement). Describe comparability of assessment methods if there is more than one group | Methods: Exposure and Outcome Assessment, Covariate Assessment |  |  |
| Bias | 9 | Describe any efforts to address potential sources of bias | Methods: Covariate Assessment, Statistical Analysis |  |  |
| Study size | 10 | Explain how the study size was arrived at | Figure 1 |  |  |
| Quantitative variables | 11 | Explain how quantitative variables were handled in the analyses. If applicable, describe which groupings were chosen and why | Methods: Statistical Analysis |  |  |
| Statistical methods | 12 | (*a*) Describe all statistical methods, including those used to control for confounding | Methods: Statistical Analysis para. 1 |  |  |
|  |  | (*b*) Describe any methods used to examine subgroups and interactions | Exploratory categorical analyses are described in Statistical Analysis. |  |  |
|  |  | (*c*) Explain how missing data were addressed | Figure 1 |  |  |
|  |  | (*d*) If applicable, explain how loss to follow-up was addressed | Not applicable |  |  |
|  |  | (*e*) Describe any sensitivity analyses | Methods: Statistical Analysis |  |  |
| **Results** | | |  |  |  |
| Participants | 13* | (a) Report numbers of individuals at each stage of study—eg numbers potentially eligible, examined for eligibility, confirmed eligible, included in the study, completing follow-up, and analysed | Figure 1 |  |  |
|  |  | (b) Give reasons for non-participation at each stage | Figure 1 |  |  |
|  |  | (c) Consider use of a flow diagram | Figure 1 |  |  |
| Descriptive data | 14* | (a) Give characteristics of study participants (eg demographic, clinical, social) and information on exposures and potential confounders | Result: Baseline characteristics of participants, Table 1 |  |  |
|  |  | (b) Indicate number of participants with missing data for each variable of interest | Figure 1 |  |  |
|  |  | (c) Summarise follow-up time (eg, average and total amount) | Methods: Study Population |  |  |
| Outcome data | 15* | Report numbers of outcome events or summary measures over time | Result, Table 1, 2, S3-S5 |  |  |
| Main results | 16 | (*a*) Give unadjusted estimates and, if applicable, confounder-adjusted estimates and their precision (eg, 95% confidence interval). Make clear which confounders were adjusted for and why they were included | Result 3.2-3.5, Fig 2-3, Table 2, S3-S5 |  |  |
|  |  | (*b*) Report category boundaries when continuous variables were categorized | Figure 2 |  |  |
|  |  | (*c*) If relevant, consider translating estimates of relative risk into absolute risk for a meaningful time period | Not applicable |  |  |
| Other analyses | 17 | Report other analyses done—eg analyses of subgroups and interactions, and sensitivity analyses | Result 3.2-3.5 |  |  |
| **Discussion** | | |  |  |  |
| Key results | 18 | Summarise key results with reference to study objectives | Discussion: The results showed that increases in the biological age indicators HD, KDM, and PA were all associated with an increase in all-cause mortality in breast cancer patients, and after adjusting for factors such as chronological age, gender and disease conditions, the results remained robust. |  |  |
| Limitations | 19 | Discuss limitations of the study, taking into account sources of potential bias or imprecision. Discuss both direction and magnitude of any potential bias | Discussion: Nevertheless, certain limitations must be recognized when interpreting our results…... |  |  |
| Interpretation | 20 | Give a cautious overall interpretation of results considering objectives, limitations, multiplicity of analyses, results from similar studies, and other relevant evidence | Discussion + Conclusion: In summary, our findings indicate that accelerated biological ageing may be associated with an increased risk of all-cause mortality in breast cancer patients, independent of chronological age, and other common cancer risk factors. This work contributes to our understanding of the biological basis of ageing and cancer, and suggests that slowing biological ageing may improve prognosis in breast cancer patients, thereby contributing to the clinical management of breast cancer. |  |  |
| Generalisability | 21 | Discuss the generalisability (external validity) of the study results | Discussion: Firstly, the predominantly non-Hispanic White composition within NHANES limits the generalizability of our conclusions to other racial populations, underscoring the necessity for multicenter validations across diverse demographic groups. |  |  |
| **Other information** | | |  |  |  |
| Funding | 22 | Give the source of funding and the role of the funders for the present study and, if applicable, for the original study on which the present article is based | Not applicable |  |  |
| *Give information separately for exposed and unexposed groups. Note: An Explanation and Elaboration article discusses each checklist item and gives methodological background and published examples of transparent reporting. The STROBE checklist is best used in conjunction with this article (freely available on the Web sites of PLoS Medicine at http://www.plosmedicine.org/, Annals of Internal Medicine at http://www.annals.org/, and Epidemiology at http://www.epidem.com/). Information on the STROBE Initiative is available at http://www.strobe-statement.org. | | | |  |  |

**Supplementary Table 2. Schoenfeld Residual Tests for the Proportional Hazards Assumption.**

***Note:*** *The proportional hazards assumption was evaluated using Schoenfeld residuals in Cox models constructed with the same covariate structure as the main survey-weighted Cox models. Model 1 adjusted for age, non-Hispanic White race/ethnicity, and PIR; Model 2 additionally adjusted for BMI, educational attainment, marital status, and smoking status; Model 3 further adjusted for hypertension, diabetes, heart failure, and CHD. Biomarker-specific P values refer to the biological age indicator in each model.* ***Abbreviations:*** *CHD, Coronary Heart Disease; PIR, Poverty Income Ratio; BMI, Body Mass Index; PA, Phenotypic Age; HD, Homeostatic Dysregulation; KDM, Klemera-Doubal Method; PH, Proportional Hazards.*

| Term | Model | Biomarker χ² | Biomarker df | Biomarker P | Global χ² | Global df | Global P |
| --- | --- | --- | --- | --- | --- | --- | --- |
| HD | Model-1 | 1.450 | 1 | 0.23 | 13.400 | 4 | 0.010 |
| HD | Model-2 | 1.040 | 1 | 0.31 | 22.400 | 12 | 0.03 |
| HD | Model-3 | 0.821 | 1 | 0.37 | 22.300 | 16 | 0.13 |
| KDM | Model-1 | 0.002 | 1 | 0.97 | 9.460 | 4 | 0.05 |
| KDM | Model-2 | 0.417 | 1 | 0.52 | 18.600 | 12 | 0.10 |
| KDM | Model-3 | 0.670 | 1 | 0.41 | 19.200 | 16 | 0.26 |
| PA | Model-1 | 3.150 | 1 | 0.08 | 11.300 | 4 | 0.02 |
| PA | Model-2 | 4.760 | 1 | 0.03 | 21.300 | 12 | 0.05 |
| PA | Model-3 | 4.500 | 1 | 0.03 | 22.700 | 16 | 0.12 |

**Supplementary Table 3. Sensitivity Analysis Using Unweighted Cox Regression Models.**

***Note:*** *Cox proportional hazards models were fitted without applying NHANES survey weights. Model 1 adjusted for age, non-Hispanic White race/ethnicity, and PIR; Model 2 additionally adjusted for BMI, educational attainment, marital status, and smoking status; Model 3 further adjusted for common chronic conditions, including hypertension, diabetes, heart failure, and CHD.* ***Abbreviations:*** *CHD, Coronary Heart Disease; PIR, Poverty Income Ratio; BMI, Body Mass Index; PA, Phenotypic Age; HD, Homeostatic Dysregulation; KDM, Klemera-Doubal Method; HR, Hazard Ratio; CI, Confidence Interval.*

| Term | Model | Sample size | Case | HR 95%CI | P |
| --- | --- | --- | --- | --- | --- |
| HD | Model-1 | 399 | 144 | 1.554 (1.304 ~ 1.853) | <0.001 |
| HD | Model-2 | 399 | 144 | 1.521 (1.263 ~ 1.832) | <0.001 |
| HD | Model-3 | 399 | 144 | 1.508 (1.245 ~ 1.825) | <0.001 |
| KDM | Model-1 | 399 | 144 | 1.018 (1.006 ~ 1.029) | 0.002 |
| KDM | Model-2 | 399 | 144 | 1.018 (1.007 ~ 1.030) | 0.002 |
| KDM | Model-3 | 399 | 144 | 1.019 (1.007 ~ 1.030) | 0.002 |
| PA | Model-1 | 399 | 144 | 1.087 (1.049 ~ 1.127) | <0.001 |
| PA | Model-2 | 399 | 144 | 1.075 (1.034 ~ 1.118) | <0.001 |
| PA | Model-3 | 399 | 144 | 1.078 (1.036 ~ 1.123) | <0.001 |

**Supplementary Table 4. Survey-Weighted Sensitivity Analysis After Excluding Outliers (>3 SD) in HD, KDM, and PA.**

***Note:*** *Outliers were defined as biomarker-specific values greater than 3 standard deviations from the mean. Survey-weighted Cox proportional hazards models were fitted after excluding outliers for each biological age indicator. Model 1 adjusted for age, non-Hispanic White race/ethnicity, and PIR; Model 2 additionally adjusted for BMI, educational attainment, marital status, and smoking status; Model 3 further adjusted for common chronic conditions, including hypertension, diabetes, heart failure, and CHD. All models accounted for the complex sampling design of NHANES using MEC examination weights, strata, and primary sampling units.* ***Abbreviations:*** *CHD, Coronary Heart Disease; PIR, Poverty Income Ratio; BMI, Body Mass Index; PA, Phenotypic Age; HD, Homeostatic Dysregulation; KDM, Klemera-Doubal Method; HR, Hazard Ratio; CI, Confidence Interval; MEC, Mobile Examination Center; NHANES, National Health and Nutrition Examination Survey.*

| Term | Model | Sample size | Case | HR 95%CI | P |
| --- | --- | --- | --- | --- | --- |
| HD | Model-1 | 394 | 141 | 1.480 (1.120 ~ 1.956) | 0.006 |
| HD | Model-2 | 394 | 141 | 1.459 (1.129 ~ 1.884) | 0.004 |
| HD | Model-3 | 394 | 141 | 1.463 (1.143 ~ 1.871) | 0.002 |
| KDM | Model-1 | 396 | 141 | 1.012 (0.998 ~ 1.026) | 0.08 |
| KDM | Model-2 | 396 | 141 | 1.014 (1.001 ~ 1.027) | 0.03 |
| KDM | Model-3 | 396 | 141 | 1.015 (1.002 ~ 1.028) | 0.02 |
| PA | Model-1 | 399 | 144 | 1.086 (1.031 ~ 1.144) | 0.002 |
| PA | Model-2 | 399 | 144 | 1.077 (1.023 ~ 1.134) | 0.005 |
| PA | Model-3 | 399 | 144 | 1.080 (1.023 ~ 1.140) | 0.006 |

**Supplementary Table 5. Survey-Weighted Sensitivity Analysis Adjusting for Estrogen Use.**

***Note:*** *Estrogen use was additionally adjusted using dr_estro. Survey-weighted Cox proportional hazards models were fitted. Model 1 adjusted for age, non-Hispanic White race/ethnicity, PIR, and estrogen use; Model 2 additionally adjusted for BMI, educational attainment, marital status, and smoking status; Model 3 further adjusted for common chronic conditions, including hypertension, diabetes, heart failure, and CHD. All models accounted for the complex sampling design of NHANES using MEC examination weights, strata, and primary sampling units.* ***Abbreviations:*** *CHD, Coronary Heart Disease; PIR, Poverty Income Ratio; BMI, Body Mass Index; PA, Phenotypic Age; HD, Homeostatic Dysregulation; KDM, Klemera-Doubal Method; HR, Hazard Ratio; CI, Confidence Interval; MEC, Mobile Examination Center; NHANES, National Health and Nutrition Examination Survey.*

| Term | Model | Sample size | Case | HR 95%CI | P |
| --- | --- | --- | --- | --- | --- |
| HD | Model-1 | 399 | 144 | 1.413 (1.102 ~ 1.813) | 0.006 |
| HD | Model-2 | 399 | 144 | 1.444 (1.144 ~ 1.822) | 0.002 |
| HD | Model-3 | 399 | 144 | 1.440 (1.143 ~ 1.813) | 0.002 |
| KDM | Model-1 | 399 | 144 | 1.014 (1.001 ~ 1.028) | 0.04 |
| KDM | Model-2 | 399 | 144 | 1.016 (1.003 ~ 1.029) | 0.02 |
| KDM | Model-3 | 399 | 144 | 1.017 (1.003 ~ 1.030) | 0.01 |
| PA | Model-1 | 399 | 144 | 1.082 (1.027 ~ 1.140) | 0.003 |
| PA | Model-2 | 399 | 144 | 1.074 (1.021 ~ 1.131) | 0.006 |
| PA | Model-3 | 399 | 144 | 1.076 (1.019 ~ 1.136) | 0.008 |

**Supplementary Table 6. Exploratory Categorical Cox Analysis Comparing the Highest Tertile With the Lower Two Tertiles.**

***Note:*** *This exploratory analysis compared participants in the highest tertile of each biological age indicator with those in the lower two tertiles combined. Survey-weighted Cox proportional hazards models were fitted. Model 1 adjusted for age, non-Hispanic White race/ethnicity, and PIR; Model 2 additionally adjusted for BMI, educational attainment, marital status, and smoking status; Model 3 further adjusted for common chronic conditions, including hypertension, diabetes, heart failure, and CHD. Three-level tertile models were not reported because the lowest tertile of PA contained no deaths, resulting in unstable Cox estimates.* ***Abbreviations:*** *CHD, Coronary Heart Disease; PIR, Poverty Income Ratio; BMI, Body Mass Index; PA, Phenotypic Age; HD, Homeostatic Dysregulation; KDM, Klemera-Doubal Method; HR, Hazard Ratio; CI, Confidence Interval.*

| Term | Comparison | Model | Sample size | Case | HR 95%CI | P |
| --- | --- | --- | --- | --- | --- | --- |
| HD | High vs Low/Mid | Model-1 | 399 | 144 | 1.895 (1.234 ~ 2.911) | 0.003 |
| HD | High vs Low/Mid | Model-2 | 399 | 144 | 1.865 (1.225 ~ 2.839) | 0.004 |
| HD | High vs Low/Mid | Model-3 | 399 | 144 | 1.870 (1.237 ~ 2.829) | 0.003 |
| KDM | High vs Low/Mid | Model-1 | 399 | 144 | 1.158 (0.711 ~ 1.887) | 0.56 |
| KDM | High vs Low/Mid | Model-2 | 399 | 144 | 1.334 (0.854 ~ 2.085) | 0.20 |
| KDM | High vs Low/Mid | Model-3 | 399 | 144 | 1.395 (0.881 ~ 2.209) | 0.16 |
| PA | High vs Low/Mid | Model-1 | 399 | 144 | 1.739 (0.563 ~ 5.369) | 0.34 |
| PA | High vs Low/Mid | Model-2 | 399 | 144 | 1.698 (0.555 ~ 5.194) | 0.35 |
| PA | High vs Low/Mid | Model-3 | 399 | 144 | 1.621 (0.533 ~ 4.931) | 0.39 |
